# Supplementary material for: Antibody stabilization for thermally accelerated deep immunostaining
Source: Nat Methods. 2022 Sep 1;19(9):1137–46. doi: 10.1038/s41592-022-01569-1 (PMC9467915; doi:10.1038/s41592-022-01569-1)
Supplement: Supplementary file 1 — Supplementary Tables 1 and 2 [file 41592_2022_1569_MOESM1_ESM.pdf]

nature portfolio
https://doi.org/10.1038/s41592-022-01569-1

Supplementary information

Antibody stabilization for thermally accelerated deep immunostaining

In the format provided by the authors and unedited

Supplementary Table 1. Antibodies, ingredients and dilutions used

Antibody target	Host	Supplier / Cat. no.	Antibody conc. (mg/ml)	Additives	ThICK compatibility
AQP1	Rb	ABclonal A4195	(uncertain)	0.05% BSA, 0.02% NaN_3, 50% glycerol, 1× PBS	✔
AQP2	Rb	ABclonal A16209	(uncertain)	0.02% NaN_3, 50% glycerol, 1× PBS	✔
Arc	Ms	Santa Cruz Biotechnology sc-17839	0.2	0.1% NaN_3, 0.1% gelatin, 1× PBS	✔
α-SMA	Ms	Progen 690001	0.05	0.5% BSA, 0.09% NaN_3, 1× PBS	✗
CR	Rb	Abcam ab702	5.860	0.09% NaN_3, “Carrier protein”, 1× PBS, pH 7.3, Van Gogh yellow diluent	✔
ChAT	Gt	Millipore AB144P	3.006	In buffer with 5mg/ml BSA, 0.2% NaN_3	✔
DBH	Rb	Sigma HPA002130	0.1	40% glycerol, 0.02% NaN_3, 1× PBS	✔
DDC	Rb	Sigma HPA017742	0.05	40% glycerol, 0.02% NaN_3, 1× PBS	✔
DLG3	Rb	Sigma HPA001733	0.1	40% glycerol, 0.02% NaN_3, 1× PBS	✔
EGR1	Ms	Santa Cruz Biotechnology sc-515830	0.2	0.1% NaN_3, 0.1% gelatin, 1× PBS	✔
c-Fos	Ms	Santa Cruz Biotechnology sc-166940	0.2	0.1% NaN_3, 0.1% gelatin, 1× PBS	✔
Gephyrin	Ms	Santa Cruz Biotechnology sc-25311	0.2	0.1% NaN_3, 0.1% gelatin, 1× PBS	✔
GFAP	Ms	Santa Cruz Biotechnology sc-58766	0.2	0.1% NaN_3, 0.1% gelatin, 1× PBS	✔
GFAP	Rt	Invitrogen 13-0030	0.351	0.1% NaN_3, 1× PBS	✔
IBA1	Rb	Wako 019-19741	0.5	1× TBS	✗
MAP2	Ms	Abcam ab11267	(uncertain)	0.097% NaN_3, 0.0268% PBS	✔
Na^+/K^+-ATPase	Rb	ABclonal A11683	(uncertain)	0.02% NaN_3, 50% glycerol, 1× PBS	✗
NeuN	Rb	Abcam ab104224	1.0	0.03% NaN_3, 50% glycerol, 1× PBS	✗
NPAS4	Rb	Invitrogen PA5-39300	1.0	50% glycerol, 150 mM NaCl, 0.02% NaN_3, 1× PBS	✔
NPHS1	Rb	ABclonal A3048	(uncertain)	0.02% NaN_3, 50% glycerol, 1× PBS	✔
NPHS2	Rb	ABclonal A17337	(uncertain)	0.02% NaN_3, 50% glycerol, 1× PBS	✔
NT5E	Rb	ABclonal A2029	(uncertain)	0.02% NaN_3, 50% glycerol, 1× PBS	✔
OLIG2	Rb	Sigma HPA003254	0.3	40% glycerol, 0.02% NaN_3, 1× PBS	✔
PDGFRA	Rb	ABclonal A2103	(uncertain)	0.02% NaN_3, 50% glycerol, 1× PBS	✔
Phospho-S6 (pSer244, pSer247)	Rb	Invitrogen 44-923G	0.25	50% glycerol, 1 mg/ml BSA, 0.05% NaN_3, 1× PBS	✔
PODXL	Rb	ABclonal A10200	(uncertain)	0.02% NaN_3, 50% glycerol, 1× PBS	✔
PSD95	Ms	NeuroMab K28/43	1.0	10 mM Tris, 50 mM NaCl, 0.065% NaN_3, pH 7.4	✔
PV	Rb	Abcam ab11427	3.568 (1.0)	3% BSA, 0.05% NaN_3, 1× PBS	✔
PV	Rb	Invitrogen PA1-933	1.0	20 mg/ml BSA, 0.1% NaN_3, 1× PBS	✔
S100b	Rb	Enzo LifeSciences ENZ-ABS307-0100	0.2	0.1 mg/ml BSA, 0.05% NaN_3, 1× PBS	✗
Synapsin I	Rb	Novus Biologicals NB300-104	0.649 (0.1)	10 mM HEPES, pH 7.5, 0.15 M NaCl, 0.1 mg/ml BSA, 50% glycerol	✔
SYNPO	Rb	ABClonal A8484	(uncertain)	0.02% NaN_3, 50% glycerol, 1× PBS	✔
SOM	Rt	Millipore MAB354	(uncertain)	Unpurified tissue culture supernatant, 0.05% thimerosal	✔
TFRC	Rb	ABclonal A5865	(uncertain)	0.02% NaN_3, 50% glycerol, 1× PBS	✔
TH	Ms	Millipore AB152	0.416	10 mM HEPES, pH 7.5, 150 mM NaCl, 0.1 mg/ml BSA, 50% glycerol	✔
TPH2	Ms	Sigma AMAb91108	1.0	40% glycerol, 0.02% NaN_3, 1× PBS	✔
VGLUT2	Ms	Sigma AMAb91081	0.5	40% glycerol, 0.02% NaN_3, 1× PBS	✔
VIP	Rb	Bioss bs-0077R	6.472 (1.0)	1% BSA, 50% glycerol, 0.09% NaN_3, “aqueous buffer”	✔

Supplementary Table 2. Comparison of large-volume tissue deep immunostaining and clearing methods

Method	Typical timeline (from tissue to imaging)
ThICK-staining using SPEARs	● 1 day of washing in PBST and synthesis of SPEARs.<br>● ThICK-staining for 16 – 72 hours.<br>● 6 hours of RI homogenization with OPTIClear (or 2 hours with OPTIClear2), or 6 hours of clearing with BABB.
vDISCO whole-body perfusion staining	● Set up transcardial perfusion.<br>● 1 day of perfusion wash with PBS.<br>● 2 days of perfusion with decolorization solution.<br>● 3 hours × 3 perfusion wash with PBS.<br>● 2 days of perfusion with decalcification solution.<br>● 3 hours × 3 perfusion wash with PBS.<br>● ½ day of perfusion with permeabilization solution.<br>● 6 days of perfusion with staining solution.<br>● 2-3 days of additional staining.<br>● 3 hours × 3 perfusion wash with washing solution.<br>● 3 hours × 3 perfusion wash with PBS.<br>● 1 day of RI homogenization with 3DISCO.<br>● Reference: http://www.discotechnologies.org/vDISCO/vDISCO_protocol.pdf
eFLASH (with SHIELD)	● 4 days of tissue preservation by SHIELD.<br>● 1 day of preparation.<br>● 3 days of active delipidation.<br>● 1 day of preparation.<br>● 1 day of eFLASH.<br>● 2 days of RI homogenization with PROTOS.<br>● Reference: https://www.biorxiv.org/content/10.1101/660373v1.full.pdf
ELAST (with SHIELD)	● 4 days of tissue preservation by SHIELD.<br>● 1 day of washing with PBST.<br>● 3 days of active delipidation.<br>● 1 day of washing with PBS with thimerosal.<br>● 2 – 3 days of washing with a washing solution.<br>● 3 – 5 days of incubation in the ELAST solution with daily exchange.<br>● 6 hours of polymerization.<br>● 4 days of clearing in clearing solution with every 2 days exchange.<br>● 2 – 4 days of washing with PBST.<br>● 1 day of blocking with blocking solution.<br>● 1 day of cyclic compression immunostaining.<br>● Overnight washing with PBST.<br>● 6 hours of antibody fixation.<br>● 2 hours × 2 washing with PBS azide<br>● 1 day of RI homogenization with PROTOS.<br>● Reference: https://www.nature.com/articles/s41592-020-0823-y#Sec2
CUBIC-HistoVIsion	● 1 day of post-perfusion fixation in PFA.<br>● 3 hours × 3 washing with PBS azide.<br>● Overnight delipidation with 0.5× CUBIC-L.<br>● 3 – 5 days of delipidation in CUBIC-L.<br>● 2 hours × 3 washing with PBS.<br>● 1.5 hours of incubation in staining buffer.<br>● 10 days – 8 weeks of staining with antibody-Fab complex in HistoVision staining buffer.<br>● 1 day of incubation at 4°C to stabilize signal.<br>● 1 hour of washing (30 minutes × 2 times).<br>● 1 day and an hour of post-fixation.<br>● Brief washing with PBS.<br>● 1 day of immersion with non-diluted CUBIC-R+(N)/(M).<br>● Reference: http://cubic.riken.jp/data/200915_Susaki%20et%20al%20(2020)_Supplementary_HV1.1.pdf
