# Supplementary material for: Antibody stabilization for thermally accelerated deep immunostaining
Source: Nat Methods. 2022 Sep 1;19(9):1137–46. doi: 10.1038/s41592-022-01569-1 (PMC9467915; doi:10.1038/s41592-022-01569-1)
Supplement: Supplementary file 2 — Reporting Summary [file 41592_2022_1569_MOESM2_ESM.pdf]

nature research

Corresponding author(s): Hei-Ming Lai, Ho Ko

Last updated by author(s): Apr 26, 2022

Reporting Summary

Nature Research wishes to improve the reproducibility of the work that we publish. This form provides structure for consistency and transparency in reporting. For further information on Nature Research policies, see our Editorial Policies and the Editorial Policy Checklist.

Statistics

For all statistical analyses, confirm that the following items are present in the figure legend, table legend, main text, or Methods section.

n/a	Confirmed	
☐	☒	The exact sample size (*n*) for each experimental group/condition, given as a discrete number and unit of measurement
☐	☒	A statement on whether measurements were taken from distinct samples or whether the same sample was measured repeatedly
☐	☒	The statistical test(s) used AND whether they are one- or two-sided<br>*Only common tests should be described solely by name; describe more complex techniques in the Methods section.*
☐	☒	A description of all covariates tested
☐	☒	A description of any assumptions or corrections, such as tests of normality and adjustment for multiple comparisons
☐	☒	A full description of the statistical parameters including central tendency (e.g. means) or other basic estimates (e.g. regression coefficient) AND variation (e.g. standard deviation) or associated estimates of uncertainty (e.g. confidence intervals)
☐	☒	For null hypothesis testing, the test statistic (e.g. F, t, r) with confidence intervals, effect sizes, degrees of freedom and P value noted<br>*Give P values as exact values whenever suitable.*
☒	☐	For Bayesian analysis, information on the choice of priors and Markov chain Monte Carlo settings
☒	☐	For hierarchical and complex designs, identification of the appropriate level for tests and full reporting of outcomes
☒	☐	Estimates of effect sizes (e.g. Cohen's d, Pearson's r), indicating how they were calculated

Our web collection on statistics for biologists contains articles on many of the points above.

Software and code

Policy information about availability of computer code

Data collection	The selective plane illumination microscopy was performed using the mesoSPIM-control (https://github.com/mesoSPIM/mesoSPIM-control) software (v0.1.5).<br>The two-photon serial tomography was performed using the ScanImage software (v5.6.1).
Data analysis	Image processing and analyses were performed using Imaris (v9), Zen Blue (v3), Fiji (ImageJ) and custom-written codes in MATLAB (R2018b, R2020b & R2021a).<br>The tractography analysis was performed using the Diffusion Toolkit (http://trackvis.org/dtk/) and the TrackVis (http://trackvis.org/) softwares.

For manuscripts utilizing custom algorithms or software that are central to the research but not yet described in published literature, software must be made available to editors and reviewers. We strongly encourage code deposition in a community repository (e.g. GitHub). See the Nature Research guidelines for submitting code & software for further information.

Data

Policy information about availability of data

All manuscripts must include a data availability statement. This statement should provide the following information, where applicable:

- Accession codes, unique identifiers, or web links for publicly available datasets
- A list of figures that have associated raw data
- A description of any restrictions on data availability

The numeric data for applicable plots are available in the Source Data file. The raw imaging data presented in this paper are too large for public deposit and will be made available upon reasonable request to the corresponding authors (H.M.L. or H.K.).

Field-specific reporting

Please select the one below that is the best fit for your research. If you are not sure, read the appropriate sections before making your selection.

☒ Life sciences ☐ Behavioural & social sciences ☐ Ecological, evolutionary & environmental sciences

For a reference copy of the document with all sections, see nature.com/documents/nr-reporting-summary-flat.pdf

Life sciences study design

All studies must disclose on these points even when the disclosure is negative.

Sample size	No statistical method was used to predetermine sample size. We decided on the sample sizes and number of experiments based on usual requirements for the specific types of comparisons. These were sufficient as evident from the respective plots in the figures and the associated statistics.
Data exclusions	No data were excluded from the analyses.
Replication	For the appropriate experiments, all replications are detaily outlined at the respective places in the manuscript. The reproducibility of experiments are evident from the respective plots showing summary statistics and error bars.
Randomization	The experiments were not randomized. Wherever applicable, we ensured identical tissue type, source, processing, and/or imaging conditions to ensure comparability.
Blinding	The Investigators were not blinded to allocation during experiments and outcome assessment. Wherever applicable, we ensured identical tissue type, source, processing, and/or imaging conditions to ensure comparability.

Reporting for specific materials, systems and methods

We require information from authors about some types of materials, experimental systems and methods used in many studies. Here, indicate whether each material, system or method listed is relevant to your study. If you are not sure if a list item applies to your research, read the appropriate section before selecting a response.

Materials & experimental systems

n/a	Involved in the study	
☐	☒	Antibodies
☒	☐	Eukaryotic cell lines
☒	☐	Palaeontology and archaeology
☐	☒	Animals and other organisms
☒	☐	Human research participants
☒	☐	Clinical data
☒	☐	Dual use research of concern

Methods

n/a	Involved in the study	
☒	☐	ChIP-seq
☒	☐	Flow cytometry
☒	☐	MRI-based neuroimaging

Antibodies

Antibodies used

Antibody (Supplier and cat. no.)
Alexa Fluor 594-conjugated donkey anti-goat IgG Fab fragments (Jackson ImmunoResearch 705-547-003)
Unconjugated donkey anti-mouse IgG Fab fragments (Jackson ImmunoResearch 715-007-003)
Alexa Fluor 488-conjugated donkey anti-mouse IgG Fab fragments (Jackson ImmunoResearch 715-547-003)
Alexa Fluor 594-conjugated donkey anti-mouse IgG Fab fragments (Jackson ImmunoResearch 715-587-003)
Alexa Fluor 647-conjugated donkey anti-mouse IgG Fab fragments (Jackson ImmunoResearch 715-607-003)
Unconjugated donkey anti-rabbit IgG Fab fragments (Jackson ImmunoResearch 711-007-003)
Alexa Fluor 488-conjugated donkey anti-rabbit IgG Fab fragments (Jackson ImmunoResearch 711-547-003)
Alexa Fluor 594-conjugated donkey anti-rabbit IgG Fab fragments (Jackson ImmunoResearch 711-587-003)
Alexa Fluor 647-conjugated donkey anti-rabbit IgG Fab fragments (Jackson ImmunoResearch 711-607-003)
Alexa Fluor 488-conjugated goat anti-rat IgG Fab fragments (Jackson ImmunoResearch 112-547-003)
AQP1 (ABclonal A4195)
AQP2 (ABclonal A16209)
Arc (Santa Cruz Biotechnology sc-17839)
α-SMA (Progen 690001)
CR (Abcam ab702)
ChAT (Millipore AB144P)
DBH (Sigma HPA002130)
DDC (Sigma HPA017742)
DLG3 (Sigma HPA001733)

	EGR1 (Santa Cruz Biotechnology sc-515830)<br>c-Fos (Santa Cruz Biotechnology sc-166940)<br>Gephyrin (Santa Cruz Biotechnology sc-25311)<br>GFAP (Santa Cruz Biotechnology sc-58766)<br>GFAP (Invitrogen 13-0030)<br>IBA1 (Wako 019-19741)<br>MAP2 (Abcam ab11267)<br>Na+/K+-ATPase (ABclonal A11683)<br>NeuN (Abcam ab104224)<br>NPAS4 (Invitrogen PA5-39300)<br>NPHS1 (ABclonal A3048)<br>NPHS2 (ABclonal A17337)<br>NT5E (ABclonal A2029)<br>OLIG2 (Sigma HPA003254)<br>PDGFRA (ABclonal A2103)<br>Phospho-S6 (pSer244, pSer247) (Invitrogen 44-923G)<br>PODXL (ABclonal A10200)<br>PSD95 (NeuroMab K28/43)<br>PV (Abcam ab11427)<br>PV (Invitrogen PA1-933)<br>S100b (Enzo LifeSciences ENZ-ABS307-0100)<br>Synapsin I (Novus Biologicals NB300-104)<br>SYNPO (ABclonal A8484)<br>SOM (Millipore MAB354)<br>TFRC (ABclonal A5865)<br>TH (Millipore AB152)<br>TPH2 (Sigma AMAb91108)<br>VGLUT2 (Sigma AMAb91081)<br>VIP (Bioss bs-0077R)<br>VGLUT2 (Sigma AMAb91081)<br>VIP (Bioss bs-0077R)
Validation	As this is an imaging method development paper, we validated all antibodies without modifications ourselves in tissue. These were based on the expected patterns of immunolabeling revealed based on biological knowledge (all figures / supplementary figures), as well as with additional comparisons to the Human Protein Atlas (Supplementary Fig. 10).

Animals and other organisms

Policy information about studies involving animals; ARRIVE guidelines recommended for reporting animal research

Laboratory animals	Male C57BL/6 and Thy1-GCaMP6f transgenic adult mice of at least 2 months old were used. The mice were provided by the Laboratory Animal Service Center of CUHK and maintained at controlled temperature (22–23 °C) with an alternating 12 h light/dark cycle with free access to standard mouse diet and water. The ambient humidity was maintained at <70% relative humidity.
Wild animals	This study did not involve wild animal.
Field-collected samples	This study did not involve field-collected samples.
Ethics oversight	All experimental procedures were approved in advance by the Animal Research Ethical Committee of the Chinese University of Hong Kong and were carried out in accordance with the Guide for the Care and Use of Laboratory Animals.

Note that full information on the approval of the study protocol must also be provided in the manuscript.
