# Supplementary figures and images for: Antibody stabilization for thermally accelerated deep immunostaining
Source: Nat Methods. 2022 Sep 1;19(9):1137–46. doi: 10.1038/s41592-022-01569-1 (PMC9467915; doi:10.1038/s41592-022-01569-1)

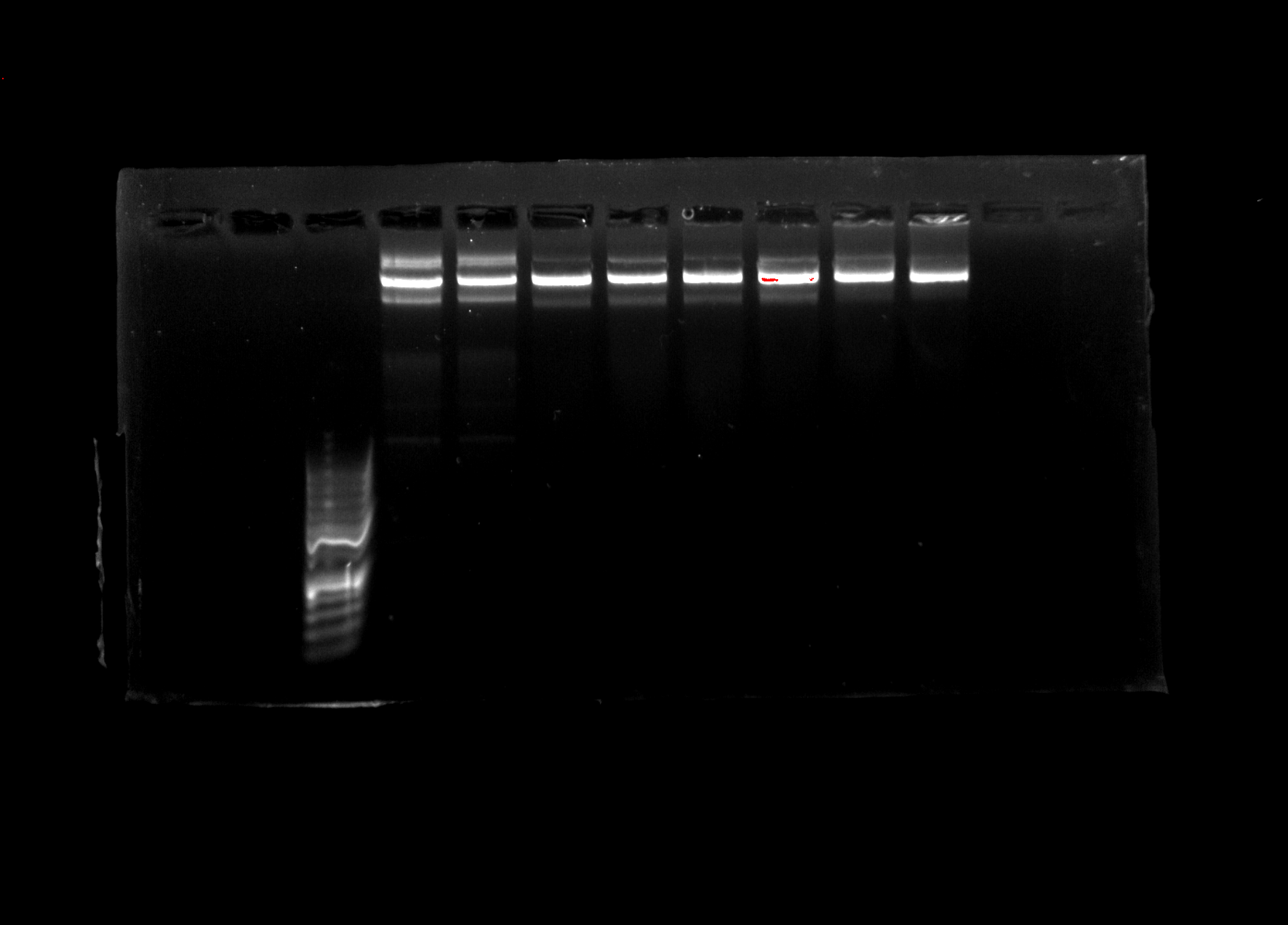

Supplement: Fig. 3j — Uncropped gel for Fig. 3j [file 41592_2022_1569_MOESM9_ESM.tif]

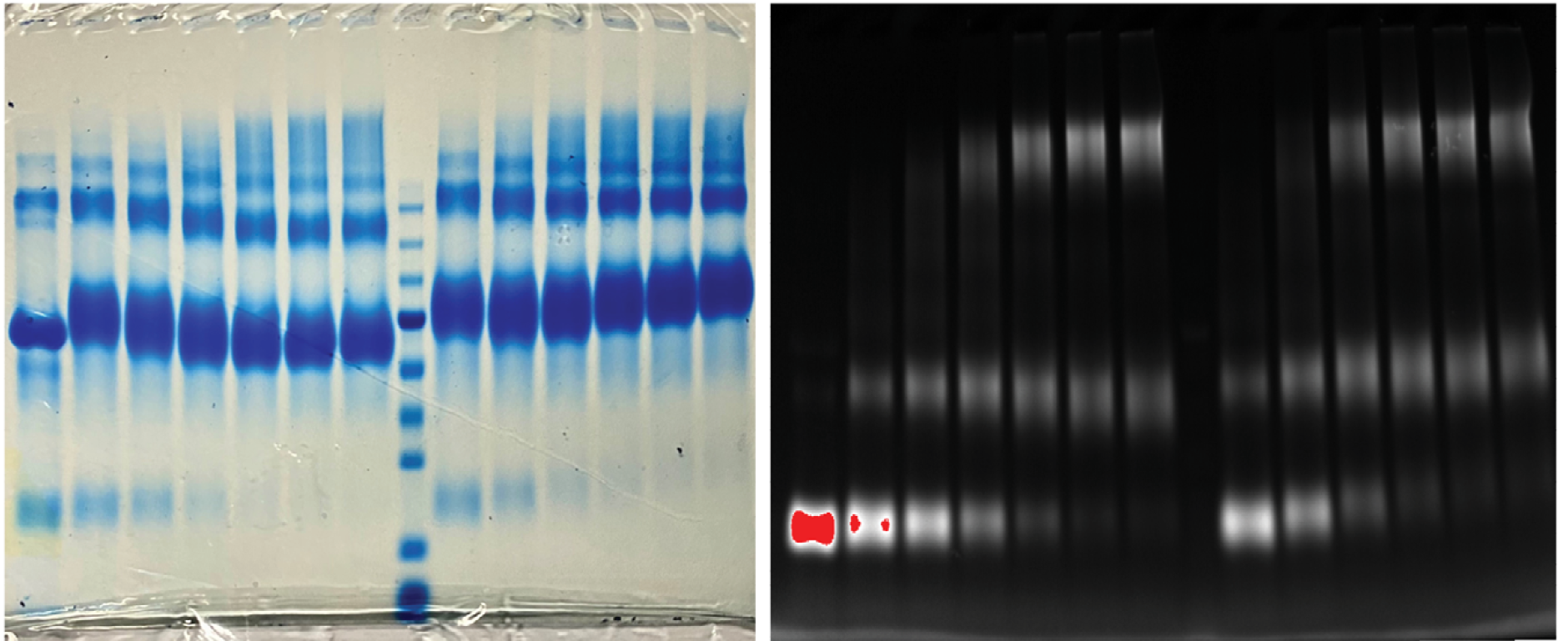

Supplement: Fig. 3i — Uncropped gel for Fig. 3i [file 41592_2022_1569_MOESM10_ESM.png]

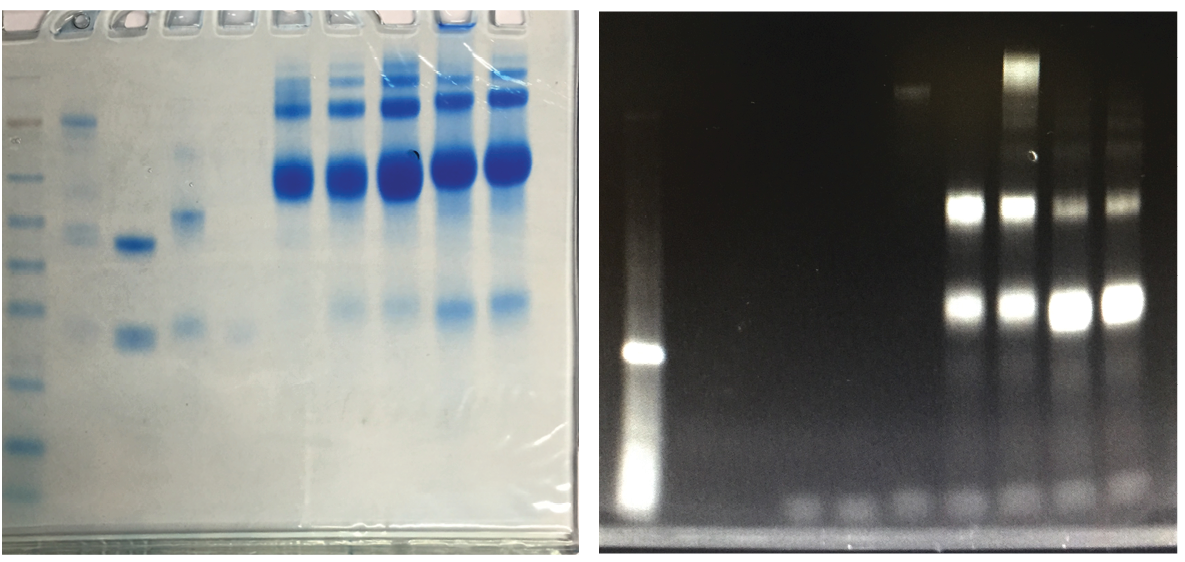

Supplement: Fig. 1f — Uncropped gel for Fig. 1f [file 41592_2022_1569_MOESM11_ESM.png]
